# DAP Kinase-Related Apoptosis-Inducing Protein Kinase 2 (DRAK2) Is a Key Regulator and Molecular Marker in Chronic Lymphocytic Leukemia

**DOI:** 10.3390/ijms21207663

**Published:** 2020-10-16

**Authors:** Katarzyna Szoltysek, Carmela Ciardullo, Peixun Zhou, Anna Walaszczyk, Elaine Willmore, Vikki Rand, Scott Marshall, Andy Hall, Christine J. Harrison, Jeyanthy Eswaran, Meera Soundararajan

**Affiliations:** 1 Translational and Clinical Research Institute, Newcastle University, Newcastle upon Tyne NE1 7RU, UK; Katarzyna.Szoltysek@newcastle.ac.uk (K.S.); carmela86@gmail.com (C.C.); elaine.willmore@newcastle.ac.uk (E.W.); andy.hall@newcastle.ac.uk (A.H.); christine.harrison@newcastle.ac.uk (C.J.H.); 2 Maria Skłodowska-Curie Institute, Oncology Center, Gliwice Branch, 02-034 Gliwice, Poland; 3 Department of Applied Sciences, Faculty of Health and Life Sciences, Northumbria University, Newcastle upon Tyne NE1 8ST, UK; 4 School of Health & Life Sciences, Teesside University, Middlesbrough TS1 3JN, UK; p.zhou@tees.ac.uk (P.Z.); v.rand@tees.ac.uk (V.R.); 5 Institute of Biosciences, International Centre for Life, Newcastle University, Newcastle upon Tyne NE1 7RU, UK; Anna.Walaszczyk@newcastle.ac.uk; 6 National Horizons Centre, Teesside University, Darlington DL1 1HG, UK; 7 Department of Haematology, City Hospitals Sunderland NHS Trust, Sunderland SR4 7TP, UK; scott.marshall@nuth.nhs.uk; 8 Newcastle University Medicine Malaysia (NUMed Malaysia), EduCity, Iskandar 79200, Johor, Malaysia

**Keywords:** CLL, DRAK2, STK17B, prognostic indicator, DAPK1

## Abstract

Chronic lymphocytic leukemia (CLL) is the most common adult leukemia in the Western World and it is characterized by a marked degree of clinical heterogeneity. An impaired balance between pro- and anti-apoptotic stimuli determines chemorefractoriness and outcome. The low proliferation rate of CLL cells indicates that one of the primary mechanisms involved in disease development may be an apoptotic failure. Here, we study the clinical and functional significance of DRAK2, a novel stress response kinase that plays a critical role in apoptosis, T-cell biology, and B-cell activation in CLL. We have analyzed CLL patient samples and showed that low expression levels of DRAK2 were significantly associated with unfavorable outcome in our CLL cohort. *DRAK2* expression levels showed a positive correlation with the expression of *DAPK1*, and *TGFBR1*. Consistent with clinical data, the downregulation of DRAK2 in MEC-1 CLL cells strongly increased cell viability and proliferation. Further, our transcriptome data from MEC-1 cells highlighted MAPK, NF-κB, and Akt and as critical signaling hubs upon *DRAK2* knockdown. Taken together, our results indicate DRAK2 as a novel marker of CLL survival that plays key regulatory roles in CLL prognosis.

## 1. Introduction

Chronic lymphocytic leukemia (CLL) represents the most common adult leukemia in the western world, accounting for ~40% of adult leukemia [1,2]. It is a malignancy of mature clonal B-lymphocytes that accumulate in the blood, bone marrow, and lymphoid tissues [2,3]. The immunophenotype of CLL is defined by the expression of B-cell markers (CD23, CD19, and weak CD20), along with the CD5 antigen and weak expression of surface membrane immunoglobulin (sIg) [4]. Despite the well-defined morphological and immunological phenotype, the clinical course and outcome of CLL are highly heterogeneous [4,5].

Unlike other B-cell malignancies, CLL is not usually associated with chromosomal translocations. Cytogenetic abnormalities, such as deletions of 13q14, 11q22-q23, and 17p13, trisomy 12 [6], and high-level expression of serum markers (CD38, Zeta-chain-associated protein kinase 70 (ZAP-70)) are critical predictors of disease progression and survival [7,8,9,10]. Unmutated *IGVH* (U-*IGVH*) cases have more aggressive disease and shorter survival times than those with mutated *IGVH* (M-*IGVH*) [11]. In addition, 15% of CLL show aberrations in a few frequently affected chromosomal regions, including *ATM* and *TP53* genes, which are correlated with disease progression and overall survival [4,12].

The precise etiology of CLL remains to be determined, although it is well-known as a lympho-proliferative disease. Patient CLL cells have been shown to be arrested in the G0/G1 phase of the cell cycle [13], resulting in low proliferation and accumulation of malignant cells that are unable to initiate their apoptotic program in blood, lymphoid tissues, and bone marrow [14]. In line with this observation, the impaired balance between pro- and anti-apoptotic stimuli is established to play key roles in chemorefractoriness and outcome of CLL [14]. Moreover, the extrinsic and the intrinsic apoptotic pathways and their regulators, such as DR4 (Death Receptor 4), TNF receptor type 1-associated death domain protein (TRADD), Fas-associated protein with death domain (FADD) and tumor necrosis factor (TNF) receptor, have been implicated in CLL development [15,16].

The death-associated protein kinase family (DAPKs) are novel stress response, serine threonine kinases, belonging to the calmodulin-regulated kinase family that have been strongly established to play a critical role in apoptosis through intrinsic, extrinsic, and p53 mediated signaling cascades [17,18]. DAPKs contain a catalytic kinase domain, death domain, ankyrin repeats, leucine zipper and a dimerization domain that are shown to regulate multiple cell processes [18]. In extrinsic apoptotic signaling, the DAPKs function downstream of receptors that respond to IFN-γ, TNF-α, FAS, and TGF-β-induction [17]. In familial, as well as sporadic CLL, loss or reduced expression of *DAPK1* due to epigenetic silencing by promoter methylation has been reported [19,20], however the precise function of DAPKs in CLL remains unclear. In addition to DAPK1, the DAPK family includes DAPK2, DAPK3, DAP kinase-related apoptosis-inducing protein kinase 1 (DRAK1), and DRAK2 [8,17].

**Figure 1:**
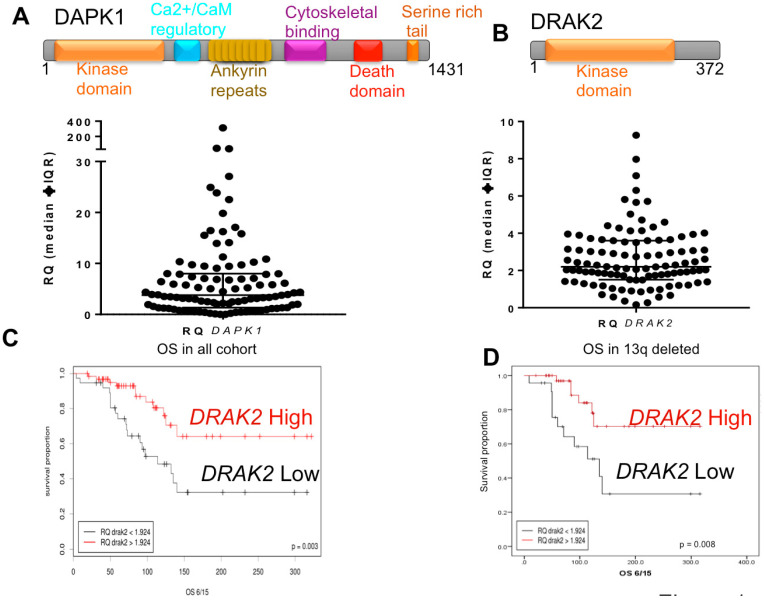
DRAK2 expression distribution and clinical impact in CLL. Domain architecture of DAPK1 and DRAK2 (Upper Panels (**A**,**B**)) and scatter plot showing the distribution of (lower panel (**A**)) *DAPK1* expression level (lower panel (**B**)) and DRAK2 expression level in a cohort of heterogeneously selected CLL cases. (**C**) Kaplan-Meier overall survival curve for patients with a high and low level of *DRAK2* expression (**D**) Kaplan-Meier overall survival curves for the 13q deleted patient subgroup with a high and low level of *DRAK2* expression.

In contrast to DAPK1, the other DAPKs and DRAKs have no death domain or calmodulin-binding site, but include a N-terminal catalytic domain and a C-terminal region that is responsible for the regulation of kinase activity and various cell signaling cascades [21] (Figure 1A,B). DRAK2 is a lymphoid kinase, expressed in T and B cells [21]. In T cells, DRAK2 is involved in setting the threshold for T cell activation, and its deficiency in T cells results in response to suboptimal stimuli, ultimately leading to a defect in survival [22]. DRAK2 and protein kinase D (PKD) form a signaling module that controls calcium homeostasis upon T-cell activation [23]. The *drak2*^−/−^ mice showed a five-fold decrease in spleen germinal centers compared to their wild-type littermates, resulting from increased B cell apoptosis [24]. Since B-cell proliferation in the germinal center drives ongoing antigen-dependent selection and generation of high-affinity class-switched plasma and memory B-cells, it is likely that DRAK2 regulates these immune response signaling processes through its apoptotic function [24]. Conversely, suppression of apoptosis through DRAK2 occurs in acute myeloid leukemia through upstream regulation of MYB oncogene [25]. Here, we have investigated the role of DAPK1 and DRAK2 in CLL biology and pathogenesis. We found only DRAK2 to have prognostic significance. Further, the influence of DRAK2 on cell survival, proliferation, and gene expression suggests a possible role for DRAK2 in CLL biology.

## 2. Results

### 2.1. Low DRAK2 Expression Level Is Associated with Shorter Overall Survival in CLL Patients

RNA expression of *DAPK1* and *DRAK2* was measured in a cohort of 102 CLL patient-derived samples (Table 1). Expression of DRAK2 and *DAPK1* showed high variability between samples (Figure 1A,B, lower panels). ROC (receiver operating characteristic) analysis was used to assign ‘High’ and ‘Low’ expression sets (Figure S1A,B). *DAPK1* RNA expression did not predict overall patient survival (OS) in our CLL cohort (Figure S1C). In contrast, *DRAK2* low-expressing patients showed a significantly shorter OS than DRAK2 high-expressing patients (*p* = 0.003), suggesting that *DRAK2* could be a new prognostic indicator in CLL (Figure 1C). *DRAK2* expression was also linked to OS among the subgroup of patients with deletions of the long arm of chromosome 13 (13q), a recognized good prognostic group in CLL (Figure 1D). It was also noteworthy that in both univariate and multivariate models, the established biomarkers (age, 17p deletion, and *IGHV* status) and *DRAK2* expression remained strong predictors of OS (Table 2). No significant correlation was identified between the expression of *DRAK2* and known clinical (age, IGHV status, mutation of *TP53*, and status of ZAP70 and CD38) and cytogenetic (13q, 11q, 17p deletions and trisomy 12) markers (Table 3; Figure S2A). CLL patients with a high white cell count or harboring 11q deletions were significantly more likely to have low *DAPK1* expression (Figure S2B), indicating a preferential downregulation of *DAPK1* within these high-risk subgroups.

### 2.2. DRAK2 Expression Level Positively Correlates with DAPK1, and TGFBR1

**Figure 2:**
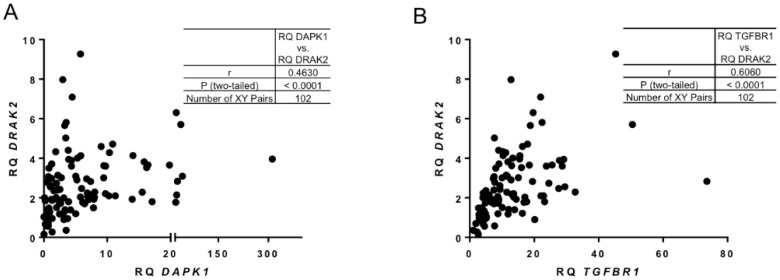
Correlation between DRAK2 and key targets of survival cellular pathways. Pearson’s correlation analysis showing strong, positive relationship between (**A**) DAPK1 and DRAK2 RNA expression and (**B**) TGFBR1 and DRAK2 RNA expression.

A very strong positive correlation was found between the expression levels of *DAPK1* and *DRAK2* (Figure 2A,B). It has been shown previously that in solid tumours transforming growth factor β Receptor I (*TGFBR1)* controls TGF-β/Smads signalling through DRAK2 [26]. Interestingly, in our CLL patient cohort, a positive correlation was detected between *DRAK2* and *TGFBR1* (Figure 2B). These data indicate a potential relationship between *DRAK2* expression and other critical regulators of cell survival in CLL.

### 2.3. Subcellular Localization of DRAK2

**Figure 3:**
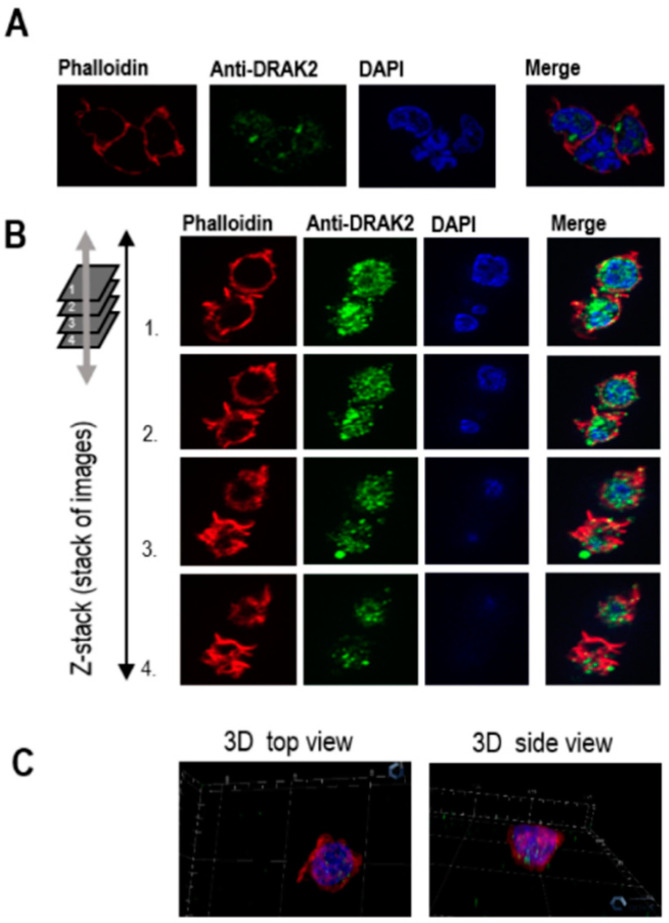
Immunofluorescence analyses of DRAK2 cellular localization in MEC-1 cells. MEC-1 cells were stained with different fluorophores to visualize cellular compartments: cytoplasm/actin filaments (visualized with phalloidin) and nucleus (visualized with DAPI). Cells were analyzed using a ZEISS Axio Imager microscope. Z1 to generate 2D view (**A**) using ZEISS Apotome that allows the collection of confocal-like “Z” stacks to project images, as shown in 3D (**B**,**C**).

Further, we investigated DRAK2 subcellular localization in CLL. Previously DRAK2 has been shown to localize within the nucleus through its ‘nuclear localization signal (NLS)’ motif in colon cancer cells and fibroblasts [27]. However, other studies have established a compelling role for DRAK2 near the plasma membrane by controlling T cell tonic signaling through the T-cell receptor directly interacting with TGFBR1, thereby regulating smad signaling [26]. When we studied the cellular localization of DRAK2 in MEC-1 cells using immunofluorescence, DRAK2 protein was found to be located within the cytoplasmic as well as nuclear compartments. However, the predominant levels of DRAK2 protein were present within the nucleus (Figure 3). Interestingly, within the cytoplasm, DRAK2 was located together with the actin filaments, indicating a possible role in cell adhesion, migration, and proliferation.

### 2.4. DRAK2 Impacts on Cell Viability and Proliferation

**Figure 4:**
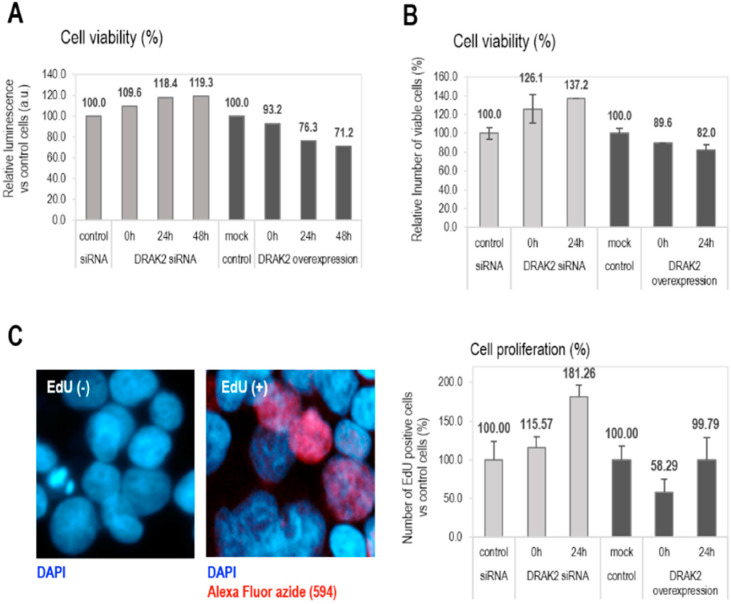
DRAK2 impact on the cellular viability (**A**,**B**) and proliferation (**C**). Cellular viability was measured using the CellTiter-Glo^®^ Luminescent Cell Viability Assay (**A**), trypan blue staining (**B**), and the cell proliferation endpoint was evaluated by cell counting following EdU (5ethynyl-2′-deoxiuridine) (**C**). All experiments were repeated 3 times, and error bars represent the standard error of the mean of 5 experiments.

The functions of DRAK2 in relation to cell survival and proliferation are reported to be cell type-dependent. For example, in triple-negative breast cancer, DRAK2 depletion clearly decreased proliferation and tumorigenic breast cancer cells’ capacity through negative regulation of TGFβR1 [26]. In contrast, DRAK2 does not appear to function as the negative regulator of TGFβ signaling in primary T cells [28]. Conversely, in pancreatic beta cells, apoptosis was induced upon overexpression of DRAK2 by interfering with Bcl-xL, Bcl-2, and Flip anti-apoptotic factors [29]. As DRAK2 expression in CLL patient samples appears to play a tumor-suppressive, pro-apoptotic role, we studied the role of DRAK2 in cell proliferation and apoptosis in CLL. To facilitate gene manipulation studies, the CLL cell line, MEC-1 was used to probe for the functional effects of DRAK2 knockdown or expression. MEC-1 cells were transfected with plasmid DNA (expressing full-length *DRAK2*) or siRNA, to obtain DRAK2 cellular variants with up- or down-regulated gene and protein expression, respectively (Figure S3). Using *DRAK2* siRNA, 60% reduction in mRNA, and 50% reduction at the protein level were achieved (Figure S3). The transfection of MEC-1 cells with *DRAK2* plasmid DNA through nucleofection resulted in 65-fold increase in mRNA and 20% increase in protein levels (Figure S3A,B). We studied the influence of DRAK2 on cell proliferation in MEC-1 cells by measuring cell viability using trypan blue staining and quantitation of ATP levels as an indicator of metabolically active, proliferating cells via a luminescent-based assay. Both tests revealed that DRAK2 downregulation increased cell viability (by 20–37%), while its overexpression reduced cell viability by 20–30% at the 24 h time point of analysis. Importantly, this tendency was sustained for 48 h when cells were analyzed using the trypan blue exclusion assay (Figure 4A,B). Furthermore, to estimate the cell proliferation rate, we analyzed the proportion of cells that incorporated EdU (5-ethynyl-2′-deoxyuridine) during DNA synthesis. Results showed that lack of DRAK2 increased cell proliferation by around 80% at 24 h, while high levels of DRAK2 temporarily suppressed cell proliferation (40% at zero time point when compared to the mock control) (Figure 4C), indicating that DRAK2 plays a role in the regulation of this cellular process.

### 2.5. Impact of DRAK2 on Gene Expression in MEC-1

**Figure 5:**
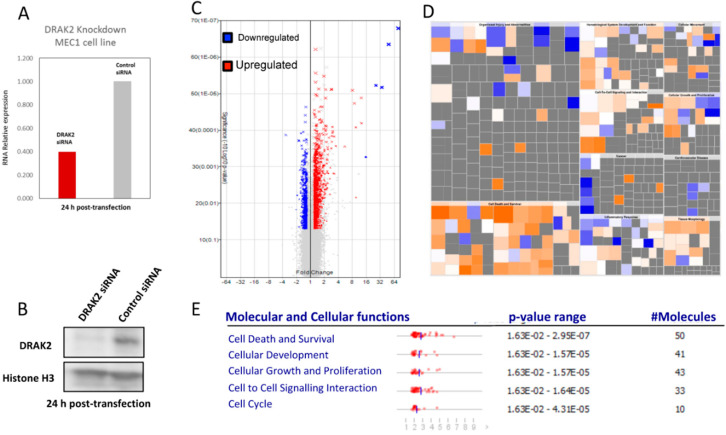
Microarray analysis reveals that MEC1 cells produce a differential gene expression signature upon DRAK2 siRNA knockdown. (**A**) DRAK2 siRNA knockdown in MEC1 cells strongly reduces DRAK2 RNA and (**B**) protein levels. (**C**) Volcano plot in which gene expression is calculated as a linear fold change relative to MEC1 cells treated with siRNA control. *p*-value is calculated by one-way between-subject ANOVA for unpaired samples. Red data points represent genes expressed > 1.2-fold and blue data points represent genes expressed < 1.2-fold in MEC1-siRNA-DRAK2 versus MEC1-siRNA-control, where *p* < 0.05. (**D**). Ingenuity’s pathway analysis of DRAK2 mediated putative genes, and biological pathways are depicted using a color-coded heatmap. The color intensity of the squares in the heatmaps reflects the strength of the absolute z-score for predictions (orange = positive, blue = negative). The size of the squares reflects the z-score values. The top five molecular and cellular functions deregulated in MEC1 cells upon DRAK2 knockdown are given in the lower panel.

To analyze changes in the gene expression due to the downregulation of *DRAK2* in MEC-1 cells and provide an overview of the affected genes and pathways, we used transcriptome arrays (Affymetrix, Human Transcriptome Array 2.0). Upon *DRAK2* knockdown (Figure 5A,B), we identified 3691 differentially expressed transcripts (FC = 1.2, FDR < 0.05, *p*-value < 0.05) (Figure 5C, Supplementary Materials Excel File S1). We performed data reduction by selecting 754 differentially expressed transcripts with a fold change of 1.5, FDR < 0.05, *p*-value < 0.05. Among the differentially expressed transcripts, 465 non-coding transcripts, including piRNA, lincRNA, lncRNA, and pseudogenes, were separated (Figure S4). Among 289 mapped transcripts, the up- and down-regulated transcripts and the cell signaling pathways influenced by them were identified using ingenuity pathway analysis (IPA). Gene enrichment within pathways that regulate cell death, growth, proliferation, hematological system, and developmental pathways were implicated (Figure 5E, Figure S5). IPA analysis further revealed key genes and pathways, including inflammatory disease, organismal injury, connective tissue disorders, and immune cell trafficking, influenced by DRAK2 downregulation (Figure S5). Among genes representative of this pathway, we identified both pro- and anti-apoptotic genes, as well as genes involved in cell proliferation. Cell death and survival emerged as the pathway most significantly deregulated upon *DRAK2* knockdown, as MAPK, NF-κB, and Akt signaling hubs were impacted (Figure S5). This study has reported, for the first time, an association between *DRAK2* expression and poor prognosis in CLL and the possible influence of DRAK2 in cell survival and proliferation of this disease.

## 3. Discussion

Recent advances in the understanding of CLL genomics have increased the availability of molecular markers for prediction of prognosis and guiding of treatment [5,30]. Assessment of the predictive molecular markers, such as *TP53* and *IGHV*, prior to treatment assignment, is a recommendation of most current guidelines for CLL management. In addition, several prognostic markers have been reported in CLL [4,8,31]. Certain consistently validated prognostic factors are now integrated into prognostic score determination. The discovery of new prognostic biomarkers is improving our understanding of the underlying biology and natural history of CLL, leading to the development of new therapeutic strategies [31]. In this study, low expression levels of *DRAK2* were significantly associated with an unfavorable outcome in CLL and specifically within the good risk (low risk) subtype with 13q14 deletions. *DRAK2* expression was found to be a significant, independent marker for OS, in multivariate analysis, even when established prognostic markers, such as *IGHV* status and 17p deletion, were included within the model. These findings highlight *DRAK2* as a possible prognostic marker with potential clinical impact. However, it remains essential to validate the impact of DRAK2 on overall survival in larger cohorts. Deletion of 13q14 is the most frequent genetic lesion in CLL, occurring in 50–60% of cases. This region contains the micro RNA cluster, including the microRNAs, *miR15A,* and *miR16A*, which inhibits the expression of key regulators of apoptosis and cell cycle in normal cells [32]. Importantly, the inclusion of a reduced level of *DRAK2* as an additional risk stratification marker will identify patients with more aggressive disease among the good risk (low risk) subtype with 13q deletions.

Although this study showed a positive correlation between *DRAK2* and *DAPK1*, *DAPK1* expression was not an indicator of prognosis, nor was it associated with other clinical markers, as reported previously. In this earlier study, increased *DAPK1* promoter methylation was observed within a family of seven individuals and 62 sporadic CLL samples, implicating that loss or reduced expression of *DAPK1* may be linked to a heritable predisposition to CLL [19]. However, reduced RNA expression of *DAPK1* was reported from a comparison of 50 unselected cells against normal CD19+ B cells, using semi-quantitative RT-PCR analysis, different from the comparison performed here. Our study compared total RNA expression from a heterogeneous cohort of 102 samples, including sporadic CLL, but no familial cases, against normal RNA from PBMC, not CD19+ B cells. While it is possible that the *DAPK1* promoter methylation mark may play a role in hereditary CLL, our study has shown that total RNA expression of *DAPK1* is not a prognostic marker in sporadic, randomly selected CLL. However, we cannot rule out this study was not adequately powered to detect significant differences to validate DAPK1 as a prognostic marker. Nonetheless, the positive correlation between *DRAK2* and *DAPK1* may be indicative of their synergistic roles in apoptosis, with *DRAK2* acting as the main inducer of cell death in sporadic CLL.

In agreement with clinical data, lack of DRAK2 increased cell viability and prolonged cell proliferation in MEC1 cells, which indicate a possible tumor suppressor role for DRAK2 in CLL. These observations are similar to previously reported results of the induction of apoptosis through DRAK2 overexpression in pancreatic beta cells upon free fatty acid stimulation [29]. Moreover, DRAK2 kinase activity-dependent apoptosis-like cell death was observed in adenocarcinoma-derived, ACL-15, cells upon UV irradiation [27]. Conversely, *Drak2^−/−^* T-cells were shown to be more susceptible to intrinsic apoptosis [33], suggesting that DRAK2 promotes survival of T-cells during clonal expansion following antigenic stimulation. Thus, the effect of DRAK2 on apoptosis appears to be pro- or anti-apoptotic, depending on cell type, which in CLL appears to be pro-apoptotic. Downregulation of DRAK2 in CLL and its association with apoptotic regulators could be events required for the leukemic cells to escape cell death, as cell viability increases due to DRAK2 downregulation in CLL.

The key apoptotic regulators, such as Bcl-2 family proteins and caspases that mediate the intrinsic as well as extrinsic apoptotic pathways, showed deregulation upon DRAK2 knockdown in our transcriptomic data. The differentially expressed genes cluster around NF-κB, ERK, PI3K, and VEGF signaling nexus (Figure S5). Indeed in CLL signaling/transcriptional pathways, including MAPK, PI3K/AKT, Wnt/β-catenin, and NF-κB that promote cell survival have been reported to be deregulated [4,34,35]. In normal B lymphocytes, the stimulation of B-cell receptor signaling triggers recruitment and activation of Syk kinase, which subsequently activates various downstream effector enzymes, including protein kinase C (PKC), phosphatidylinositol 3-kinase (PI3K), and phospholipase Cγ2 [36]. These signaling complexes regulate key downstream pathways, such as AKT, ERK, c-Jun NH_2_-terminal kinase, and p38, that determine the B-cell fate [4,5,34]. Several studies have suggested that AKT activation promotes CLL cell survival following BCR engagement [37]. In the context of T-cells, TCR activation determines the accumulation of DRAK2 in PKC and MAP kinase-dependent manner. However, a prominent feature of CLL B-cells is the low level of surface IgM expression and distinct B-cell receptor (BCR) signaling compared to normal B cells with low-level IgM expression [38]. Further, dysregulation of the BCR signaling in CLL is characterized by constitutively active phosphorylation of certain kinases, such as Lyn and Syk that also triggers cell survival pathways. A number of the above-mentioned pathways could therefore be possibly involved in DRAK2 mediated cell survival and apoptotic functions that we have observed here. However, systematic validation of the impact of DRAK2 on the common apoptotic and the survival pathway members and their functional implications is necessary to fully elucidate the molecular mechanism of DRAK2 in CLL.

Although the role of DRAK2 as a tumor suppressor is evident from the CLL patient cohort and cell line experiments, the mechanism through which these functions are implemented needs to be investigated. Further delineation of DRAK2 mediated signaling pathways that regulate cell proliferation and pro-survival pathways, such as AKT/ERK, Syk/Lyn, and Wnt/β-catenin, in patient-derived CLL cells from both treatment naïve and refractory cases will allow us to better understand the role of DRAK2 in CLL biology and assist in the identification of new drug targets in this disease.

## 4. Materials and Methods

### 4.1. Patients and Cell Isolation

Peripheral blood samples (*n* = 102) from CLL patients were collected in EDTA-coated tubes. Informed consent was obtained in accordance with the Declaration of Helsinki, and with approval from the NHS Research Ethics Committee. These samples were processed immediately using Lymphoprep (Axis Shield, Cambridgeshire, UK). CLL patient sample collection, processing, and storage were carried out in accordance with the regulations of the Human Tissue Act 2004 (UK). The research was conducted using samples obtained through the Newcastle Biobank (17/NE/0361).

### 4.2. Experimental Model and Nucleofection

Human chronic B-cell leukemia MEC-1 cell line (DSMZ no: ACC 497) was purchased from DSMZ and used as an experimental model. MEC1 is an EBV transformed line and thus proliferates in culture, making it useful for studies including gene manipulation. Although the MEC-1 line does not recapitulate all the features of CLL, it was used as a model to examine the function of DRAK2 in human B cells. The cells were mostly used for experiments between passages 11 and 19 after thawing fresh cells. Cells were grown in IMDM medium supplemented with 10% fetal bovine serum (Gibco) at 37 °C in a humidified 5% CO_2_ atmosphere. Human B cell Nucleofector Kit™ and Nucleofector™ Platform (Lonza) with Nucleofector Program X-001 was used in gene manipulation studies. Cells were transiently transfected with 20 nM siRNA (Qiagen, Hilden, Germany) or 5 µg plasmid DNA (Addgene, Watertown, MA, USA) to obtain DRAK2 knockdown or overexpression, respectively. Please refer to Supplementary Materials Table S2 for antibodies. Cells were transfected for 18 h using sequences specific for DRAK2 gene (mix of four different siRNA: 5′-TGGTTAGACAATGTATATCAA-3′, 5′-CTGAGATAGCTCTGCAGATAA-3′ 5′-CACGAGATTGCTGTGCTTGAA-3′ and 5′-AGCATATATGTTGTTAACTCA-3′; upper strands only) or negative control siRNA (Qiagen); in overexpression experiments, cells were transfected for 24 h using control (empty/mock) pEGFP-N1-FLAG vector (Addgene; 60360) or pEGFP-N1-FLAG vector with DRAK2 corresponded to full-length cDNA sequence (ORF of serine/threonine kinase 17b (STK17B)). DRAK2 encoding cDNA was cloned into pEGFP-N1-FLAG using BamH1 and Mlul. Transfection efficiency, in this case, was verified based on the level of EGFP positive cells.

### 4.3. RNA Extraction and qPCR

Total RNA was extracted from PBMCs using the GeneJet RNA purification kit (Thermo Scientific). Concentration and purity of the total RNA samples were measured using a NanoDrop ND-1000 Spectrophotometer. cDNA was synthesized with a high-Capacity cDNA synthesis kit (Life Technologies) following the manufacturer’s protocol. Relative quantification of *DAPK1*, *DRAK2*, and *TGFBR1* RNA expression was performed by qPCR based on TaqMan chemistry and carried out in an Applied Biosystems Viia 7 Real-Time PCR System (Applied Biosystems, Foster City, CA, USA). *GAPDH* was used as a reference for the normalization of qPCR and a pool of five RNA samples extracted from peripheral blood of five healthy donors was used as a calibrator. All qPCR reactions were performed in biological duplicate and technical triplicates to assess data reproducibility.

TaqMan probes used for this study were: Hs02758991_g1 (*GAPDH*), Hs00892459_m1 (*DRAK2*), Hs00234480_m1 (*DAPK1*), Hs00610320_m1 (*TGFBR1*) (Supplementary Materials Table S1).

### 4.4. Expression and Data Analysis

Calculations for RNA expression were made using the comparative CT (2^–ΔΔCt^) method. The potential diagnostic value of *DAPK1* and *DRAK2* RNA expression was assessed by a ROC curve to identify optimal cut-off points (Figure S1). *DAPK1* and *DRAK2* expression was then split at this diagnostic cut-off, and the resulting dichotomous variable was included in a Kaplan-Meier overall survival (OS) analysis. Prognostic variables associated with OS were identified through univariate analysis by Cox regression models. The hazard ratio (HR) and 95% confidence interval (CI) were calculated using Cox regression models. The independent prognostic variables associated with OS were confirmed by multivariate analysis using the Cox proportional hazards model. Any differences between OS curves were evaluated using the log-rank test. In the correlation analysis reported, all variables were treated as categorical. All statistical calculations were performed using GraphPad Prism 6, and SPSS Statistics v23 (see Supplementary Materials). Pearson’s test was used to assess the correlation between categorical variables. Spearman’s test was used to assess the correlation between continuous variables. The Mann-Whitney U test was used to compare data in subgroups. Differences were considered statistically significant when *p*-value was <0.05.

### 4.5. Cell Viability Assay

Cell viability was determined with the application of the CellTiter-Glo^®^ Luminescent Cell Viability Assay (Promega), according to the manufacturer’s instructions. Cells with different status of DRAK2 were analyzed at different time points from the beginning of the transfection process; the zero h time point is regarded to be the optimal time point for incubation of cells with siRNA or plasmid DNA (equivalent to 18 and 24 h, respectively). Luminescence was calculated using a FLUOstar Omega microplate reader and MARS Data Analysis Software (BMG Labtech, Ortenberg, Germany). The relative number of viable cells compared to the control MEC-1 cellular variant was determined.

### 4.6. EdU Cell Proliferation Assay

Cell proliferation was evaluated by cell counting following EdU (5-ethynyl-2′-deoxiuridine) incubation with the application of Click-iT^®^ EdU Imaging Kit (C10339, Invitrogen, Carlsbad, CA, USA) according to the manufacturer’s instructions. Briefly, in order to downregulate or overexpress the level of DRAK2, MEC1 cells were subjected to siRNA or plasmid DNA transfection (via nucleofection) protocol and then incubated with 5 uM EdU (soluble in DMSO) conjugated with AlexaFluor^®^ azide (AlexaFluor^®^, Thermo Fisher Scientific, Waltham, MA, USA) to determine the number of proliferating cells. As a negative control, all cellular variants (with different DRAK2 status) were also treated with a corresponding amount of the solvent (DMSO). After 18 or 24 h (for DRAK2 knock-down or overexpression, respectively) from the transfection start point, cells were fixed in 0.4% formaldehyde/PBS, permeabilized with 0.3% Triton X100/PBS and smeared onto glass microscopic slides. Cells were washed twice with 3% BSA/PBS and once with PBS. Slides were mounted with DAPI containing HardSet medium (Vectashield; DAKO) and analyzed using an epifluorescence microscope (ZEISS Axio Imager Z1) and 5–6 images (objective 40×) of each experimental variant, corresponding to at least 100 cells, were taken. Numbers of positively stained (red) and negative (blue) cells/nuclei were calculated using ImageJ software, and relative proliferation values (percentage of EdU positive cells) were determined.

### 4.7. Trypan Blue Assay

Cell viability and proliferation were also evaluated by cell counting following the trypan blue (Gibco) exclusion assay. Cells were counted at two time points: (i) after 24 h (zero time point) or (ii) 48 h (24 h time point) of continuous incubation with siRNA or plasmid DNA. The relative numbers of viable cells (percentage) compared to the control MEC-1 cellular variant was determined.

### 4.8. Apoptosis Induction

Apoptosis was assessed using PE Annexin V Apoptosis Detection Kit I (BD Pharmingen, San Diego, CA, USA) according to the manufacturer’s instruction. All analyses were performed with the application of viable cells. Assays were conducted 18 h post silencing and 24 h post overexpression. Cells were stained with viability marker (7-AAD) and Annexin V PE-conjugate. Twenty thousand events were counted in each sample and the relative percentage of cells in the different fraction was determined—quadrants representing healthy cells, necrotic cells, and early and late apoptotic cells.

### 4.9. Gene Expression Level Analyses: Microarrays and qRT-PCR

Total RNA was extracted from 1 × 10^6^ MEC-1 cells using the RNeasy Mini Kit (Qiagen) and treated in solution with TURBO DNA-free™ Kit (Invitrogen, Renfrew, UK) or on-column with DNase, using the RNase-Free DNase Set (Qiagen). Quality and quantity of obtained RNA were analyzed by the Eukaryote Total RNA Nano Assay on an Agilent 2100 Bioanalyzer (RNA integrity numbers >8) and NanoDrop ND-1000 Spectrophotometer, respectively. Transcriptome profiling was performed by AROS Applied Biotechnology (Aarhus, Denmark) using GeneChip Human Transcriptome Array 2.0. Array data processing, background correction, normalization, and quality control checks were performed using the Transcriptome Analysis Console (TAC) Software (Thermo Fisher Scientific, UK). Differentially expressed genes (in comparison to the control sample) were selected. Genes were considered as significantly changed when: (i) ANOVA *p*-value < 0.05 and (ii) fold change (linear) was <−1.5 or > 1.5. Pathway analysis was performed using Ingenuity Pathway Analysis (IPA) Software (Qiagen, Manchester, UK). The data discussed in this article will be deposited in NCBI Gene Expression Omnibus and accessible through GEO Series accession number.

For qRT-PCR analyses, purified RNA was subjected to a two-step RT-PCR reactions. cDNA synthesis was carried out with the High-Capacity cDNA synthesis kit (Life Technologies); amounts corresponding to 50 ng of RNA were used as templates. Transcript levels of selected genes were quantified by qRT-PCR (Applied Biosystems Viia 7 Real-Time PCR System) using SYBR Green dye or TaqMan probes. All reactions were carried out in triplicate, and expression levels were normalized according to the *GAPDH* housekeeping gene. Sequences of primers and TaqMan probes used are presented in Supplementary Materials Table S1.

## 5. Conclusions

In summary, we have shown for the first time that *DRAK2* expression is linked to Overall Survival (OS) in CLL patients, potentially through regulation of cell survival and proliferation critical for leukemic cell fate. The transcriptomic data indicates the possible signaling molecules, including NFkB, BCL-2, and ERK to participate in DRAK2 mediated functions. It will therefore be informative to further investigate molecular signaling pathways and mechanistic details of DRAK2 mediated action in CLL to completely validate as a target for monitoring CLL pathogenesis and progression.

## Figures and Tables

**Table 1:** Clinical and molecular characteristics of the CLL cohort used in this study.

Characteristics	N	Percentage
Age ≥ 65	65	63.7
Sex: Male	71	69.6
**Binet stage *n* = 90**		
A	49	54.4
B	16	17.8
C	25	27.8
***IGHV* status *n* = 63**		
Mutated	38	60.3
Unmutated	25	39.7
**TP53 status *n* = 100**		
Mutated	12	12
Unmutated	88	88
**Cytogenetics *n* = 101**		
13q	62	61.4
11q	20	19.8
12+	6	5.9
17p	8	7.9
Normal karyotype	24	23.8
**ZAP70 status *n* = 19**		
>20%	9	47.4
<20%	10	52.6
**CD38 status *n* = 36**		
>20%	13	36.1
<20%	23	63.9
**Treatment status *n* = 102**		
Treated	35	34.3
Untreated	67	65.7

**Table 2:** Overall Survival analyses of the established CLL biomarkers (age, 17p deletion, and *IGHV* status) and the *DRAK2* expression.

Covariate	HR	STD Err	*p*-Value	Conf. Interval		HR	STD Err	*p*-Value	Conf. Interval	
	Univariate Analysis					Multivariate Analysis				
**Age > 70 years**	1.05	0.02	0.014	1.01	1.091	1.094	0.026	0.001	1.04	1.151
**17p status**	3.681	0.542	0.016	1.273	10.65	3.188	0.619	0.06	0.947	10.729
**IGHV status**	3.093	0.418	0.007	1.363	7.02	5.514	0.473	0.0003	2.183	13.925
**DRAK2 expression**	0.343	0.379	0.005	0.163	0.722	0.428	0.427	0.04	0.185	0.987

**Table 3:** Correlation analysis between relative expression of *DRAK2* and common clinical and molecular biomarker in CLL.

		13q Status	11q Status	+12 Status	17p Status	Age	Gender	ZAP70	CD38	IGHV	TP53 Mut
**RQ DRAK2**	Pearson r	0.014	−0.178	−0.064	−0.075	−0.033	−0.068	−0.267	−0.129	−0.111	−0.028
	p	0.892	0.075	0.524	0.456	0.741	0.495	0.270	0.453	0.387	0.783
	N	101	101	101	101	102	102	19	36	63	100

## Supplementary Materials

The following are available online at https://www.mdpi.com/1422-0067/21/20/7663/s1, Figure S1: ROC analysis and impact of DAPK1 on CLL overall survival. Figure S2: DRAK2 and DAPK1 expression distribution across relevant clinical and molecular CLL subtypes. Figure S3: Characterisation of experimental model. Figure S4: Coding and uncoding transcripts deregulated upon DRAK2 knockdown in MEC1 cells. Figure S5: DRAK2 mediated signalling networks implicated in various biological processes and disease conditions. Table S1: TaqMan probes and primers sequences used in the PCR analyses. Table S2: Antibodies used in Western blot and immunofluorescence analyses.
