# Supplementary material for: DAP Kinase-Related Apoptosis-Inducing Protein Kinase 2 (DRAK2) Is a Key Regulator and Molecular Marker in Chronic Lymphocytic Leukemia
Source: Int J Mol Sci. 2020 Oct 16;21(20):7663. doi: 10.3390/ijms21207663 (PMC7593912; doi:10.3390/ijms21207663)
Supplement: Supplementary file 1 [file ijms-21-07663-s001.zip › New folder (2)/DRAK2 CLL paper_Supplementary_230920_submission.docx]

**Supplementary material**

**Supplementary Figure S1:** ROC analysis and impact of DAPK1 on CLL overall survival

**(A)** ROC analysis showing the best cut off to define low- and high- DAPK1 expression. **(B)** ROC analysis showing the best cut off to define low- and high- DRAK2 expression. **(C)** Kaplan-Meier overall survival curve for patients with high and low level of DAPK1 expression.

**Supplementary Figure S2:** DRAK2 and DAPK1 expression distribution across relevant clinical and molecular CLL subtypes

**(A)** Box plot graphs showing distribution of *DRAK2* and *DAPK1* expression level in 13q, 11q, 17p and +12 cytogenetic CLL subgroups; **(B)** Box plot graphs showing distribution of *DRAK2* and *DAPK1* expression level in CLL subgroups showing different IGHV status, low and high white cells count, different ages groups and treatment status.

**Supplementary Figure S3:** Characterisation of experimental model

**(A)** - qRT-PCR analysis of DRAK2 expression level (data normalized to the level of GAPDH) in MEC-1 cells upon DRAK2 knock down or up regulation. Shown results are averages derived from 3 technical replicates; bars indicate standard deviation.
**(B)** - Western-blot analysis of DRAK2 protein level and its densitometry-based measurement (level of DRAK2 protein was normalized to the level of Histone H3). **(C)** - Efficiency of cell transfection (overexpression experiments) observed as occurrence of an EGFP-positive cells.

**Supplementary Figure S4:** Coding and uncoding transcripts deregulated upon DRAK2 knockdown in MEC1 cells

Microarray analysis-MEC1 siRNA DRAK2 vs MEC1 siRNA control

**
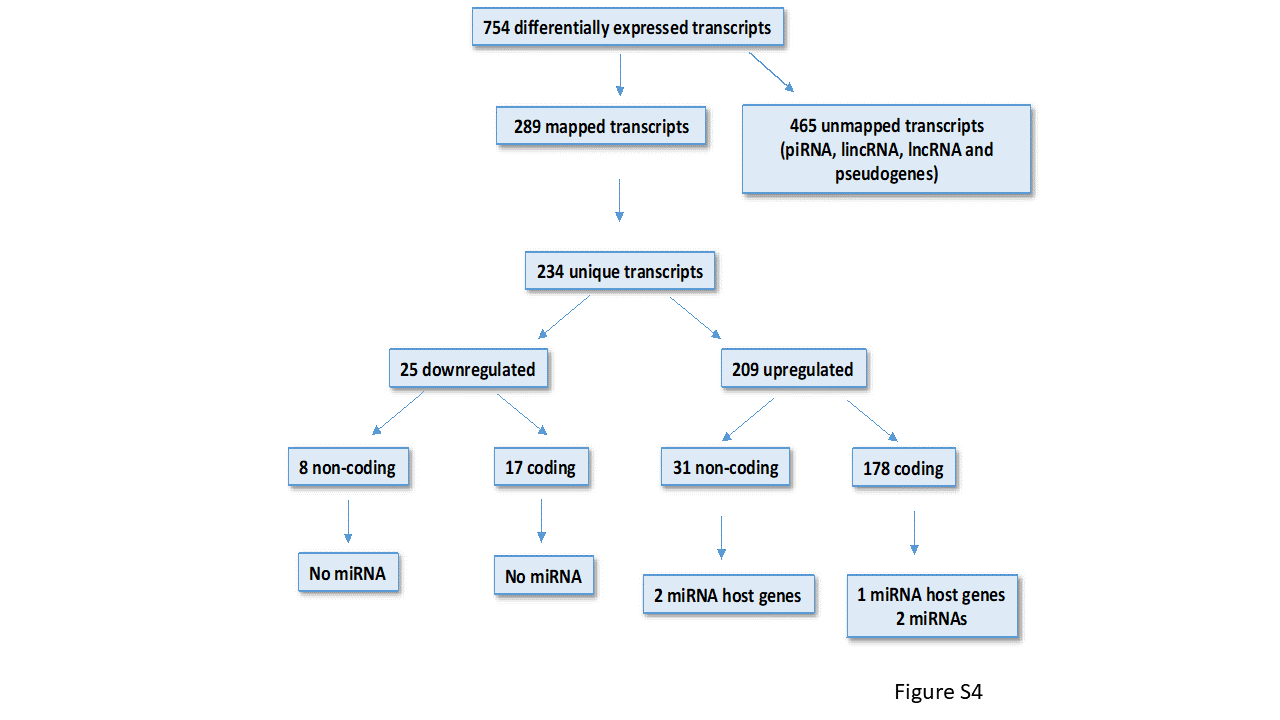
**

Flow diagram showing the 754 deregulated transcripts identified by Transcriptome Analysis Console (TAC) analysis exhibiting at least 1.5-fold change in expression with P<0.05. Transcripts were both coding and non-coding with a major, preferential induction of upregulated targets.

**Supplementary Figure S5: DRAK2 mediated signalling networks implicated in various biological processes and disease conditions.**

The differentially expressed gene candidates and the signalling networks impacted upon DRAK2 knocked down MEC1 cells in comparison with MEC1 siRNA control are shown below. Ingenuity Pathway Analysis of top networks with the highest score based on the number of differentially regulated transcripts and the p-value range.

The red colour indicates the upregulated and blue the down regulated genes.

The shapes in the figure depicts the following. Round-membrane associated proteins, Diamond- Genes involved in gene regulation control, Horizontal Oval and Vertical Oval represents transcription factor/ mRNA binding and Corepressor receptor activity genes respectively. Inverse triangle indicate enzymes and square represents signalling receptor /cytokine binding genes. Rectangles depict GPCR activity and chemokine ligand binding genes. Upright and inverted trapezoid represents oxidoreductase activity genes and microRNAs respectively.

**Supplementary Figure 5A**


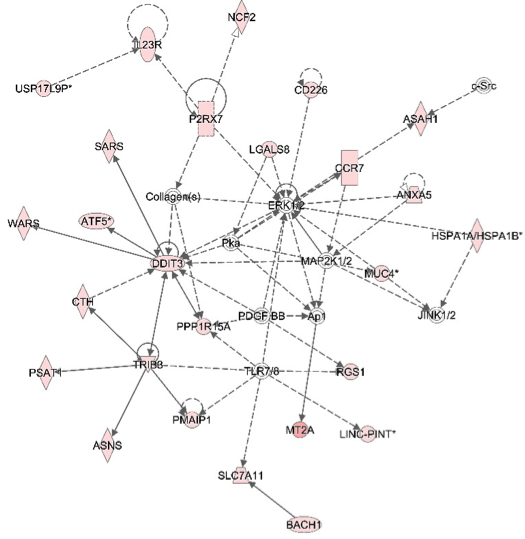


Figure S5A

The signalling network with NF-kB as central node.

**Supplementary Figure 5B**

The signalling network with AKT and VEGF as central nodes.

**Supplementary figure S5C**

Network here shows Proteins and signalling network involved in Cell Death and survival, Connective Tissue Disorders, Inflammatory Disease. ERK1/2 is identified as central node. The signalling network with MAP Kinase as central node.

**Supplementary Figure 5B**


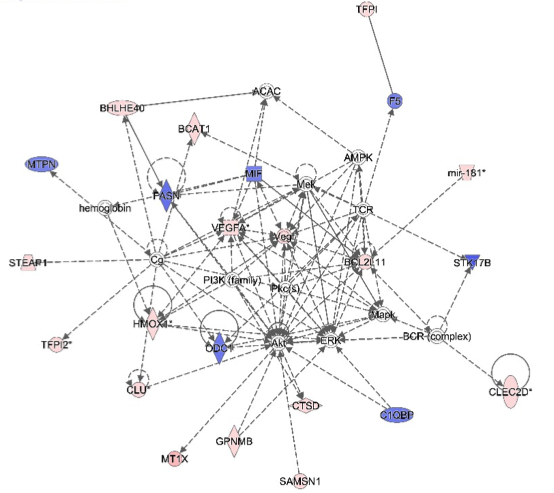


Figure S5B

Network here shows proteins and signalling network involved in Cancer, Organismal injury and reproduction system diseases. AKT and VEGF proteins are identiﬁed as central nodes.

**Supplementary Figure 5C**


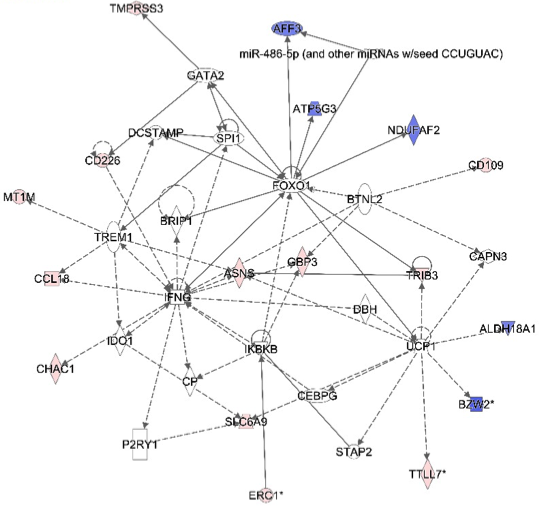


Figure S5C

Network here shows proteins and signalling network involved in Cellular growth and proliferation, Haematological system development and function. FOXO1 and FNG are identified as possible nodes.

**Supplementary Figure 5D**:


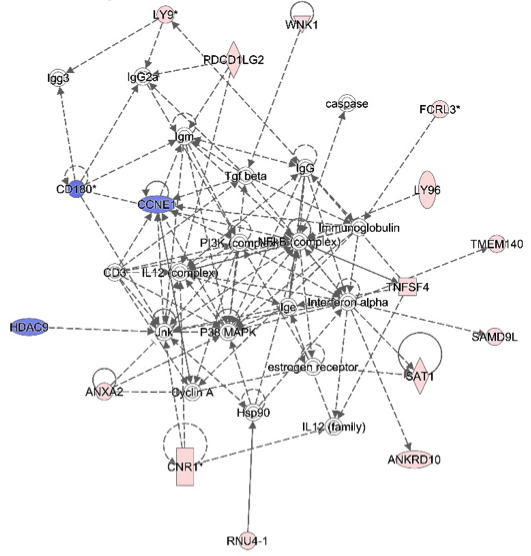


Figure S5D

Network here shows Cell-To-Cell Signaling and Interaction, Hematological System Development and Function, Immune Cell Trafficking. NFkB and p38 MAPK are the central nodes

**Supplementary Methods and Materials**

***Protein level estimation using Western blot*:** Protein expression levels were confirmed using the Western blot protocol. Whole cell protein lysates were extracted from CLL cells (derived from peripheral blood of CLL patients) and MEC-1 cells by Pierce™ RIPA buffer (Thermo Scientific) supplemented with Halt™ Protease and Phosphatase Inhibitor Single-Use Cocktail (Thermo Scientific). Protein was quantified by the Pierce™ BCA Protein Assay Kit (Thermo Scientific). Equal amounts of protein from each sample were loaded in 8% SDS-polyacrylamide electrophoresis gel, transferred to polyvinylidene difluoride (PVDF) membrane (Millipore) and blocked with 5% skimmed milk in Tris-Buffered Saline (TBS) with 0.1% TWEEN 20 (Sigma-Aldrich). Then the PVDF membrane was incubated overnight at 4°C with the primary antibody in blocking reagent (antibodies recognized total protein forms) or 5% BSA in TBS with 0.1% TWEEN 20 (phosphorylated forms of proteins), followed by the corresponding secondary antibody for 1 hour at room temperature. PVDF membrane was then developed with Amersham ECL Western Blotting detection reagents (GE Healthcare) and detected by Amersham Hyperfilm ECL (GE Healthcare). To reprobe with a different antibody, PVDF membrane was stripped with ReBlot Plus Strong Antibody Stripping Solution (Millipore). Protein bands were semi-quantified by densitometry with ImageJ software. β-Actin or Histone H3 was used as an endogenous control for normalisation of protein expression. All antibodies used in this study are listed in the Supplement File Table S2.

***Statistical analyses:*** All statistical calculations were performed using GraphPad Prism 6 and SPSS v23. All experiments were performed at least in triplicate; presented data are shown as mean values ±SD. P value was calculated with application of Student *t*-test or ANOVA (microarray analyses). Cox regression models were implemented to test the independent (univariate) and simultaneous (multivariate) effect of factors on overall survival.

***Immunocytofluorescence analyses:*** 1x10^6^ cells were transferred from cell culture and washed with PBS by centrifugation. Cells were fixed in 0.4% formaldehyde/PBS for 20 minutes, permeabilized with 0.3% Triton X100/PBS and smeared onto glass microscope slides. Cells were washed twice with PBS/0.1% Tween-20, followed by blocking with 5% BSA in PBS/0.1% Tween-20 for 1 hour. Slides were incubated overnight in humidified chambers with Anti-DRAK2 (Sigma Aldrich or Abcam, diluted 1:100) in blocking solution at 4^0^C. Slides were washed three times with PBS/0.1% Tween-20 for 5 minutes and incubated for 1 hour in humidified chambers with secondary antibody (anti-rabbit IgG) conjugated with AlexaFluor 488 followed by PBS/0.1% Tween-20 rinse. Slides were incubated with CytoPainter Phalloidin-iFluor 680 Reagent (Abcam) for 20 minutes at room temperature, rinsed with PBS and mounted with DAPI containing HardSet medium (Vectashield; DAKO). Slides were analysed with an epifluorescence microscope (ZEISS Axio Imager Z1). ZEISS Apotome 2 was used for **structured illumination to produce a confocal-like 3D image** (several cells were analysed at 8 depths, every 1 um, to create z-stacks for images that can be rotated in three dimensions for better visualisation of antigen localization). See supplementary table 2 for antibodies used.

**Flow cytometry-based analyses:** Cell cycle phase distribution and apoptosis induction analyses were performed with application of FACS Canto II cytometer and analysed with FACS Diva 8.0 Software (Becton Dickinson).

***Cell cycle phase distribution*:** Cells were centrifuged for 2 minutes at 200 x g, washed in PBS, fixed in 70% ethanol and stored at -20°C. Fixed cells were washed twice in PBS and re-suspended in PBS containing 0.2 mg/ml RNase A for 15 minutes at room temperature. Cells were stained with 50 μg/ml propidium iodide and analysed immediately with flow cytometer. Thirty thousand events were counted in each sample and relative percentage of cells in different fraction was determined.

**Supplementary Table 1: TaqMan probes and primers sequences used in the PCR analyses**

| TaqMan  Probes | **Gene name** | **Assay ID (Thermo Fisher Scientific)** |
| --- | --- | --- |
|  | *DRAK2/STK17B* | Hs 00892459_m1 |
|  | *DAPK1* | Hs 00234480_m1 |
|  | *TGFBR1* | Hs 00610320_m1 |
|  | *GAPDH* | Hs 99999905_m1 |

**Supplementary Table 2: Antibodies used in Western blot and immunofluorescence analyses**

| Antibody | Details/Cat.No |
| --- | --- |
| Anti-DRAK2 | Abcam/ab8419 |
| Anti-DRAK2 | Sigma Aldrich/PRS2149 |
| Anti-Histone H3 | Cell Signaling/4499 |
| Anti-β -actin | Abcam/ab49900 |
| Anti-rabbit IgG (HRP) | Cell Signaling/7074 |
| Anti-mouse IgG (HRP) | Cell Signaling/7076 |
| Goat anti-rabbit IgG (Alexa Fluor 488) | Invitrogen/A11008 |
| Goat anti-rabbit IgG (Alexa Fluor 488) | Invitrogen/A11001 |
